# Decreased Global EEG Synchronization in Amyloid Positive Mild Cognitive Impairment and Alzheimer’s Disease Patients—Relationship to *APOE ε4*

**DOI:** 10.3390/brainsci11101359

**Published:** 2021-10-16

**Authors:** Una Smailovic, Charlotte Johansson, Thomas Koenig, Ingemar Kåreholt, Caroline Graff, Vesna Jelic

**Affiliations:** 1 Division of Clinical Geriatrics, Center for Alzheimer Research, Department of Neurobiology, Care Sciences and Society, Karolinska Institutet, 14152 Huddinge, Sweden; Vesna.Jelic@ki.se; 2 Department of Clinical Neurophysiology, Karolinska University Hospital, 14186 Huddinge, Sweden; 3 Division of Neurogeriatrics, Center for Alzheimer Research, Department of Neurobiology, Care Sciences and Society, Karolinska Institutet, 14152 Huddinge, Sweden; charlotte.johansson@ki.se (C.J.); caroline.graff@ki.se (C.G.); 4 Clinic for Cognitive Disorders, Karolinska University Hospital, 14186 Huddinge, Sweden; 5 Translational Research Center, University Hospital of Psychiatry, University of Bern, 3012 Bern, Switzerland; thomas.koenig@upd.unibe.ch; 6 Aging Research Centre, Karolinska Institutet and Stockholm University, 17165 Solna, Sweden; ingemar.kareholt@ki.se; 7 School of Health and Welfare, Aging Research Network—Jönköping (ARN-J), Institute for Gerontology, Jönköping University, 55111 Jönköping, Sweden; 8 Unit for Hereditary Dementia, Karolinska University Hospital-Solna, 17176 Solna, Sweden

**Keywords:** quantitative electroencephalography, apolipoprotein E, Alzheimer’s disease, mild cognitive impairment, amyloid pathology

## Abstract

The apolipoprotein E (*APOE*) *ε4* allele is a risk factor for Alzheimer’s disease (AD) that has been linked to changes in brain structure and function as well as to different biological subtypes of the disease. The present study aimed to investigate the association of *APOE ε4* genotypes with brain functional impairment, as assessed by quantitative EEG (qEEG) in patients on the AD continuum. The study population included 101 amyloid positive patients diagnosed with mild cognitive impairment (MCI) (*n* = 50) and AD (*n* = 51) that underwent resting-state EEG recording and CSF Aβ42 analysis. In total, 31 patients were *APOE ε4* non-carriers, 42 were carriers of one, and 28 were carriers of two *APOE ε4* alleles. Quantitative EEG analysis included computation of the global field power (GFP) and global field synchronization (GFS) in conventional frequency bands. Amyloid positive patients who were carriers of *APOE ε4* allele(s) had significantly higher GFP beta and significantly lower GFS in theta and beta bands compared to *APOE ε4* non-carriers. Increased global EEG power in beta band in *APOE ε4* carriers may represent a brain functional compensatory mechanism that offsets global EEG slowing in AD patients. Our findings suggest that decreased EEG measures of global synchronization in theta and beta bands reflect brain functional deficits related to the *APOE ε4* genotype in patients that are on a biomarker-verified AD continuum.

## 1. Introduction

Alzheimer’s disease (AD) is the most common form of dementia with distinct neuropathological changes and a heterogeneous clinical presentation. The disease is neuropathologically characterized by the gradual accumulation of amyloid-β 42 (Aβ42) and hyperphosphorylated tau proteins in the brain tissue in the form of senile plaques and neurofibrillary tangles, respectively [1,2]. However, AD can present with different clinical symptomatology in its typical and atypical forms, depending on whether memory or other specific cognitive domains are affected [3]. These individual differences in the vulnerability of the human brain to the AD-associated neuropathology depend on several elements, including genetic predisposition, environmental effect, and lifestyle factors [4,5,6].

Even though an increasing number of genetic variants have been associated with AD [6], the apolipoprotein ε4 (*APOE*) genotype remains the main known genetic risk factor for the more common sporadic form of AD [7,8]. The *APOE* gene encodes three alleles—*ε2*, *ε3,* and *ε4*—with an allele-frequency ranging from 4–8%, 72–87%, and 9–19%, respectively, among the healthy population worldwide [7]. An extensive meta-analysis demonstrated that heterozygous and homozygous *APOE* *ε4* carriers have a 1.1–5.6- and 2.2–33.1-fold increased risk, respectively, for developing AD compared to non-carriers [7]. The mechanism by which *APOE ε4* exerts these effects remains to be fully elucidated; however, its physiological role in generating and maintaining synapses suggests impaired synaptic connections as the contributing factor, especially in the event of brain injury [8,9]. *APOE ε4* has been additionally related to increased Aβ accumulation [10], altered Aβ clearance [11], interaction with tau protein [12], and neuroinflammation in brain tissue [13].

Previous studies have shown that *APOE ε4* affects brain functional activity and connectivity, even before the presence of Aβ pathology in the brain tissue [14,15]. These findings highlight the relationship between genetic factors and brain functional changes in subjects that are at increased risk for dementia. The heritability and genetic origin of brain functioning have been particularly emphasized by studies employing electroencephalography (EEG) [16,17,18]. 

EEG is a non-invasive diagnostic procedure that registers brain activity in real time [19] and that can be further used to quantify and localize changes in the temporal and spatial organization of brain neuronal networks [20]. Quantitative EEG (qEEG), therefore, provides objective and comprehensive assessment of brain functional activity commonly analyzed across its slow (delta and theta) and fast (alpha and beta) frequency bands that are thought to reflect distinct physiological processes [19]. Individual differences in these EEG signals were shown to have strong genetic origin [18], including a high concordance of EEG signals between identical twins [16,21,22].

A number of studies have assessed qEEG changes in patients with cognitive impairment and AD, coming from a standpoint that AD is a disease of synaptic failure [23,24] and that EEG is the only available method that can directly mirror synaptic activity with a millisecond time resolution [25]. The most consistent qEEG findings in patients on the clinical AD continuum include generalized EEG-slowing and reduced EEG synchronization in fast frequency bands [26,27,28,29,30,31]. These qEEG changes have been additionally related to the severity of cognitive impairment [32,33,34,35], future cognitive decline and progression to dementia [27,36,37], as well as with a cerebrospinal fluid (CSF) profile of AD biomarker changes [38,39]. 

Several studies have further addressed a potential relationship between qEEG changes and the *APOE* status in patients with AD; however, the results have been conflicting thus far, reporting accentuated EEG slowing in *APOE ε4* carriers [40,41], more severe EEG slowing in *APOE ε4* non-carriers [42], and no differences in relation to the *APOE* status [43]. Notably, these studies involved patients whose diagnoses were based on clinical criteria and without the biomarker evidence of AD pathology, which, considering the pathophysiological heterogeneity of cognitive disorders, may have affected the interpretation of results. 

The aim of the present study was to investigate relationship between *APOE* genotype and brain functional impairment in memory clinic patients on a biomarker-verified AD continuum. We employed two qEEG measures of global power and synchronization that have been utilized previously in studies on AD and cognitive disorders [28,29,34,38]. Our hypothesis was that the *APOE* genotype has an intrinsic effect on brain oscillatory activity in MCI and dementia patients that have positive biomarkers of AD pathology. Furthermore, we investigated whether comprehensive qEEG analyses, including measures of both power and synchronization of EEG oscillations, may provide complementary information on these brain functional changes. 

## 2. Materials and Methods

### 2.1. Study Population

The study population included in total 101 patients clinically diagnosed with MCI (*n* = 50) according to the Winblad et al., 2004 criteria [44] or with AD (*n* = 51) according to the ICD-10 criteria [45]. All patients were recruited at the Clinic for Cognitive Disorders, Karolinska University Hospital Huddinge, Stockholm, Sweden and underwent comprehensive clinical assessment, computed tomography (CT) and/or magnetic resonance brain imaging (MRI), resting-state EEG recording, CSF sampling, analysis of AD biomarkers (Aβ42, phospho tau (*p*-tau), and total tau (t-tau)), and *APOE* genotyping of peripheral blood-DNA. 

All diagnostic tests were part of the baseline cognitive assessment and patients were, therefore, drug naïve with respect to AD medication. The severity of cognitive impairment was, among other tests, assessed using the Mini-Mental State Examination (MMSE) [46]. The exclusion criteria included the presence of any significant psychiatric or neurological comorbidity, history of brain trauma, use of antiepileptic or neuroleptic medications, and any other dementia diagnosis. 

Patients were stratified into three groups based on their *APOE* status and numbers of *ε4* alleles including *APOE ε4* non-carriers (*n* = 31), *APOE ε4* heterozygous carriers—one allele (*n* = 42) and *APOE ε4* homozygous carriers—two alleles (*n* = 28). All MCI and AD patients included in this study were amyloid positive according to their CSF Aβ42 levels and, therefore, meet the research and biomarker criteria for Alzheimer’s disease continuum [3,47]. MCI and AD patients were pooled in the following analyses due to a limited number of patients that prevented separate analyses of diagnostic groups. The number of MCI and AD patients within each *APOE* genotype group are presented in Table 1.

### 2.2. CSF Analysis

CSF samples were obtained by a standard lumbar puncture procedure between the L3/L4 or L4/L5 intervertebral space. All CSF samples were sampled using a 25-gauge needle, collected in 12 mL polypropylene tube, centrifuged at 1000 rpm (10 min), and frozen at −70 °C. Conventional CSF markers of AD pathology, including Aβ42, *p*-tau, and t-tau protein concentrations, were analyzed at the clinical chemistry laboratory Karolinska University Hospital, Huddinge, using xMAP technology and the INNO-BIA AlzBio3 kit (Innogenetics) [48]. Patients on the AD continuum present with decreased CSF Aβ42 levels, which is thought to reflect increased deposition and reduced clearance of Aβ42 into the CSF [49]. The clinical cut-off for pathological CSF levels, defined by Karolinska University Hospital laboratory, was CSFAβ42 levels <550 ng/L. MCI and AD patients with pathological CSF Aβ42 levels were defined as amyloid positive in the present study.

### 2.3. Resting-State EEG Recordings and Analyses

All patients underwent resting-state EEG recordings at the Department of Clinical Neurophysiology within 6 months of the baseline clinical assessment. EEGs were recorded on the Nervus system (NicoletOne EEG Reader v5.93.0.424, Natus NicoletOne, Pleasanton, CA, USA) using 21 electrodes and standard electrode placement according to the 10/20 system. The electrode impedances were kept below 5 kU. The EEGs were recorded with a sampling rate of 256 Hz and band-pass filter between 0.5 and 70 Hz. 

All digital EEG files were exported for research purposes. The EEG recordings were first assessed for any physiological and non-physiological artifacts as well as periods of drowsiness by visual inspection. Artifacts and periods of drowsiness were then removed by manual selection and the rejection of referred EEG segments. Eye movements and electrocardiographic artifacts were additionally removed using independent component analysis algorithm. The EEGs were first segmented in the 2-s artifact-free epochs. Next, Fast Fourier Transform (FFT) was performed on all available 2-s EEG epochs in order to translate EEG data into the frequency domain. 

The present study employed two global frequency domain qEEG measures that have been validated previously in the context of cognitive impairment and AD [28,36,38] named GFP and GFS. GFP is a single and generalized measure of the strength of scalp potential fields [25,50]. It can be further employed in the frequency domain where its formula corresponds to the root mean of squared spectral amplitudes across all EEG channels and, therefore, summarizes global EEG power across pre-defined frequency bands [25,28,38]. GFS is a global measure of brain functional connectivity that reflects the phase synchrony of EEG oscillations across all electrode sites [51]. In more detail, FFT analysis of EEG epochs yields, for a given frequency, sine and cosine coefficients of all electrodes that can be entered into a sine–cosine diagram. Multichannel-EEG recording, at a given time point or time epoch, can, therefore, be presented as a cloud spread of electrode points in the sine–cosine diagram. For the computation of GFS at a given frequency, these points were submitted to a principal component analysis (PCA): GFS is then defined as the ratio of the two resulting PCA eigenvalues. The more of the variance is explained by the first principal component, the more of the electrode entry points approximate the straight line and the closer GFS is to 1, which would imply that all EEG sources observable at the given frequency oscillated either in phase or in antiphase. GFS is, therefore, a measure of a common phase of EEG oscillations at all electrode sites at a certain frequency point and can obtain a value between 0 and 1. Both GFP and GFS measures were averaged over all EEG epochs and conventional frequency bands were defined as delta (1–3.5 Hz), theta (4–7.5 Hz), alpha (8–11.5 Hz), and beta (12–19.5 Hz). EEG preprocessing and quantitative analysis were performed in Brain Vision Analyzer, version 2.0, software (Gilching, Germany).

### 2.4. Statistical Analysis

Statistical analyses were performed in SPSS (version 26, IBM, New York, NY, USA) and STATA (version 16.1, StataCorp LLC, College Station, TX, USA). Demographics (age, gender, and education) and MMSE levels were compared between *APOE ε4* non-carriers, heterozygous, and homozygous carriers using the Independent-samples Kruskal–Wallis Test for continuous variables and Chi-Square test for categorical variables. 

qEEG measure GFP in all frequency bands was transformed with zero-skewness natural logarithmic transformation in order to obtain non-skewed data distributions. In the figures, the differences in GFP and GFS across *APOE* groups were presented using the original untransformed data. P-values are based on one-way analysis of variance (ANOVA) on the transformed variables. Since the assumption of homogeneity of variance was not met for all GFP/GFS variables (Levene’s test < 0.05), the *p*-values from Welch’s ANOVA were reported. Welch’s ANOVA was run separately for GFP/GFS measures in each frequency band. The level of statistical significance was *p* < 0.05.

## 3. Results

### 3.1. Demographics and Clinical Characteristics

Demographics and clinical characteristics of our study population and across the three *APOE ε4* genotype groups (non-carriers, carriers with one allele, and carriers with two alleles) are presented in Table 1. There were no statistically significant differences in age (*p* = 0.766), education (*p* = 0.631), or distribution of females versus males (*p* = 0.447) between the three groups. The *APOE ε4* non-carriers, heterozygous, and homozygous carriers did not differ significantly in the global severity of cognitive impairment as assessed by MMSE (*p* = 0.092). 

### 3.2. Relationship between Global EEG Power and APOE Genotype in Amyloid Positive MCI and AD Patients

**Figure 1:**
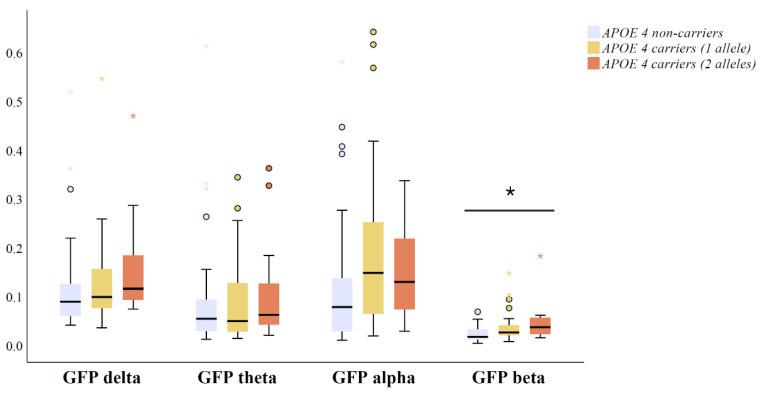
Differences in qEEG measures of global field power (GFP) between *APOE ε4* carriers with two alleles (*n* = 28), carriers with one allele (*n* = 42) and non-carriers (*n* = 31) including CSF Aβ42 positive (<550 ng/L) MCI and AD patients. The original data on GFP are presented as the median (solid line), interquartile range (box), and minimum and maximum values (whiskers) across four conventional frequency bands. *p*-Values are based on ANOVA over the three genotype groups using GFP measures transformed with zero skewness natural log-transformation; * *p* < 0.05. Outlier and extreme values are denoted as circles and stars, respectively. Abbreviations: ANOVA = analysis of variance; and *APOE* = apolipoprotein E.

GFP medians and interquartile ranges in four conventional frequency bands across *APOE ε4* genotype groups are presented in Figure 1. One outlying GFP alpha data point from an *APOE ε4* heterozygous carrier was excluded from the analysis (GFP alpha > 2 µV). There was a statistically significant gradient-like increase in GFP beta (*p* = 0.001) in amyloid positive *APOE ε4* heterozygous and homozygous carriers compared to non-carriers (Figure 1). Interestingly, there were no significant differences in GFP in delta (*p* = 0.065), theta (*p* = 0.491), or alpha bands (*p* = 0.084) between amyloid positive *APOE* genotype groups (Figure 1).

### 3.3. Relationship between Global EEG Synchronization and APOE Genotype in Amyloid Positive MCI and AD Patients

**Figure 2:**
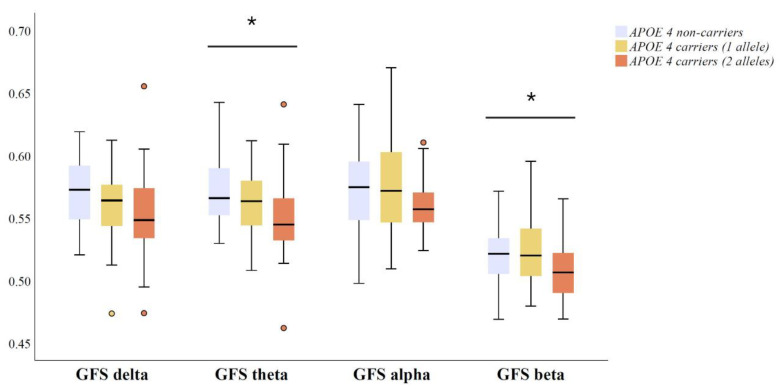
Differences in qEEG measures of global field synchronization (GFS) between *APOE ε4* carriers with two alleles (*n* = 28), carriers with one allele (*n* = 42) and non-carriers (*n* = 31), including CSF Aβ42 positive (<550 ng/L) MCI and AD patients. The original data on GFS are presented as the median (solid line), interquartile range (box), and minimum and maximum values (whiskers) across four conventional frequency bands. *p*-Values are based on ANOVA over the three genotype groups; * *p* < 0.05. Outlier and extreme values are denoted as circles and stars, respectively. Abbreviations: ANOVA = analysis of variance; and *APOE* = apolipoprotein E.

GFS medians and interquartile ranges in four conventional frequency bands across *APOE* genotype groups are presented in Figure 2. Amyloid positive *APOE ε4* non-carriers, heterozygous and homozygous carriers differed in GFS measure in theta (*p* = 0.041) and beta bands (*p* = 0.036). That is, *APOE ε4* carriers exhibited lower EEG synchronization in theta and beta bands compared to non-carriers (Figure 2). Even though a trend was observed, the differences in GFS in delta (*p* = 0.090) and alpha (*p* = 0.079) band did not reach statistical significance (Figure 2).

## 4. Discussion

The main finding of the present study was that a decrease in qEEG measures of global brain synchronization in theta and beta bands is associated with the presence of an *APOE ε4* genotype in MCI and AD patients with amyloid biomarker changes indicative of AD pathology. Additionally, amyloid positive *APOE ε4* carriers exhibited an increase in global EEG power in beta band compared to non-carriers. Our study, therefore, demonstrated an association between *APOE ε4* genotypes and intrinsic brain activity and connectivity, as assessed by qEEG analyses, in patients with cognitive dysfunction that are on a biomarker-verified AD continuum. 

Decreased global EEG synchronization in theta and beta bands may reflect more severe brain functional impairment in patients that are on the AD continuum and carry the *APOE ε4* allele compared to non-carriers. Reduced EEG synchronization in fast frequencies, including alpha and beta bands, has been supported by numerous studies involving MCI and AD patients and is consistent across various qEEG measures of brain functional connectivity [29,43,52,53,54]. The level of decrease in alpha and beta synchronization has been additionally associated with the disease severity [29,34,35], performance on neuropsychological tests [31], and AD biomarker changes in CSF, including a decrease in Aβ42 and increase in *p*-tau and t-tau levels [38]. Importantly, beta activity has been related to a number of cognitive processes including working and episodic memory [55,56,57], language processing [58], visual perception [59], decision making [60], and attentional processes [61,62]. Therefore, alterations in the idle, “resting-state” beta activity and synchronization may be associated with inadequate engagement of beta rhythms during various cognitive tasks and, consequently, with impairment of multiple cognitive domains that is characteristic of AD dementia. 

We further report that Aβ positive *APOE ε4* carriers exhibit lower GFS in the theta band compared to *APOE ε4* non-carriers. There is an overlap between the brain areas that are typically affected in amnestic syndromes and brain regions that are thought to generate theta rhythm including hippocampus and entorhinal cortex [19]. In this context, decreased global theta synchronization in amyloid positive *APOE ε4* carriers may reflect a limbic predominant pathology associated with clinical presentation of typical AD. A recent meta-analysis that addressed heterogeneity of biologically subtypes of AD reported several characteristics of limbic-predominant AD, including amnestic syndrome, late-onset sporadic presentation, and the presence of the *APOE ε4* genotype [63]. 

Several previous studies have assessed the relationship between *APOE* status and changes in EEG power across conventional frequency bands. Most of them reported a pattern of qEEG changes that are characteristic for “EEG slowing”, including increase in delta and theta and decrease in alpha and beta power and/or amplitude in MCI and AD patients that were *APOE ε4* carriers compared to non-carriers [40,41,64,65]. One of the studies reported no changes in EEG power with respect to the *APOE ε4* status; however, it included a single measure of EEG power ratio and a modest number of study participants [43]. 

In contrast, a more recent large-scale study by de Waal and colleagues included 320 AD patients and 246 healthy controls and demonstrated higher EEG delta and theta and lower alpha power in AD patients that were *APOE ε4* non-carriers compared to carriers, indicating more pronounced EEG slowing in *APOE ε4* non-carriers [42]. Our results may provide some insights on these contradictory reports. We reported an increase in beta power (and a non-significant trend towards an increase in alpha and delta power) as the only significant change in global EEG power measures in amyloid positive *APOE ε4* heterozygous and homozygous carriers compared to non-carriers, partly supporting recent reports from de Waal and colleagues that demonstrated less severe EEG slowing in *APOE ε4* carriers. Increases in EEG beta power may appear counterintuitive in the context of qEEG changes in AD; however, these results stem from MCI and AD patient groups that have not been contrasted to healthy controls, i.e., the comparison was made to *APOE ε4* negative and amyloid positive MCI and AD patients. In that regard, increased EEG beta power may reflect a mechanism of brain functional compensation in amyloid positive *APOE ε4* carriers that are at increased risk for future cognitive deterioration [8,66]. Overall, our results suggest that the main drawback of previous studies is lack of biological and pathological characterization of the study population. 

The increases in delta and theta power in previous studies may reflect a non-specific qEEG alterations in cognitively impaired *APOE ε4* carriers, driven by a number of subjects without underlying AD pathology. As it is known from the literature and clinical practice, a number of MCI subjects will progress to other types of dementias, including dementia with Lewy bodies (DLB), Parkinson disease dementia (PDD), and vascular dementia or remain cognitively stable over time [67,68], while a substantial proportion of clinically diagnosed AD patients may have underlying vascular, mixed, or tau-related pathologies [69,70,71]. Interestingly, *APOE ε4* has also been associated with increased risk for Lewy Body disease, including DLB and PDD [72], as well as with vascular dementia [73]. 

It would be of interest to further investigate these associations in cognitively healthy *APOE ε4* carriers with and without evidence of AD pathology. These studies would elucidate whether *APOE ε4* carriers exhibit disturbances in brain functional connectivity even before the clinical appearance of symptoms and amyloid pathology as reported by some of the functional MRI studies [14,15]. A limitation of the present study is that a rather conservative and binary cut-off for CSF amyloid positivity was used for the stratification of both MCI and AD patients. These biomarker cut-offs were initially derived from AD patient cohorts in order to clinically support a diagnosis of AD dementia, and a less stringent cut-off may be required for mild cognitive disorders [47,74]. 

Modification of brain activity and functional connectivity by the *APOE* genotype could further aid selection of qEEG parameters that may contribute to the prediction of AD or even different clinico-biological AD subtypes. In the quest for such sensitive biomarkers, qEEG analysis could be extended to sleep EEG and different provocation methods during standard EEG recordings, such as hyperventilation and photostimulation. Interestingly, Ponomareva and colleagues reported that cognitively healthy relatives of AD patients that were *APOE ε4* carriers had higher occurrence of synchronous delta and theta as well as sharp-waves during hyperventilation condition compared to relatives that were *APOE ε4* non-carriers [65]. This study indicated that an EEG activation paradigm may be required to accentuate early brain functional impairment in cognitively healthy patients who are at a genetically increased risk for dementia.

In conclusion, our study demonstrated that decreased EEG global synchronization in theta and beta bands reflect brain functional deficits related to the *APOE ε4* genotype in patients with a cognitive dysfunction and biomarker-verified AD pathology. Future studies on novel qEEG approaches and both established and new AD risk genes are required to demonstrate whether qEEG parameters could serve as potential endophenotypes for cognitive disorders due to AD. 

## Figures and Tables

**Table 1:** Demographic and clinical characteristics in *APOE ε4* non-carriers and carriers.

	*APOE ε4* Non-Carriers	*APOEε4* Heterozygous Carriers (One Allele)	*APOEε4* Homozygous Carriers (Two Alleles)	*p*-Value
N (total)	31	42	28	
MCI	13	22	15	
AD	18	20	13	
Age (years)	65.03 ± 9.17	65.79 ± 8.54	64.04 ± 5.31	0.766
Sex (M/F)	15/16	17/25	9/19	0.447
Education (years)	11.97 ± 3.80	12.39 ± 3.80	12.68 ± 3.42	0.631
MMSE ^a^	24.73 ± 4.32	26.71 ± 2.76	25.11 ± 4.14	0.092

Data presented as the means ± standard deviation. ^a^ Missing values for MMSE variables: one patient per each *APOE ε4* group. Independent-Samples Kruskal–Wallis Test and Chi-Square test over the three *APOE* genotype groups as appropriate. AD = Alzheimer’s disease; *APOE* = Apolipoprotein E; CSF = cerebrospinal fluid; MCI = mild cognitive impairment; and MMSE = Mini Mental State Examination.

## Data Availability

The datasets used and/or analyzed during this study are available from the study’s senior and corresponding authors on reasonable request.
